# Quantitative assessment of pelvic reduction using a pretensioned external fixator in unstable pelvic injuries: a retrospective study

**DOI:** 10.1007/s00590-026-04906-8

**Published:** 2026-09-03

**Authors:** Queipo-de-Llano Alfonso, Mariscal Lara Jorge, Leiva Gea Antonio, Delgado Rufino Francisco de Borja

**Affiliations:** 1 Faculty of Medicine, University of Málaga, Málaga, Spain; 2 Instituto de Investigación Biomédica de Málaga, Málaga, Spain

**Keywords:** Pelvic fractures, External fixation, Pre-tensioned fixator, Majeed score, Orthopaedic trauma

## Abstract

**Introduction:**

Conventional anterior external fixation provides effective stabilization in pelvic injuries but has limited ability to reduce and control posterior pelvic ring displacement, particularly in type C fractures. Although several anterior fixation systems capable of posterior compression have been described, many remain complex and impractical in emergency settings.

**Purpose:**

To evaluate the effectiveness of a pretensioned pelvic external fixator (PPEF) as a reduction tool in unstable pelvic fractures across different clinical scenarios.

**Methods:**

A retrospective study was conducted including 28 patients treated with PPEF between 2012 and 2024. In 24 cases, the device was used as emergency stabilization, and in 4 cases as an adjunct or intraoperative reduction tool. Pelvic reduction was assessed using pre- and postoperative CT scans. Anterior and posterior diastasis, as well as vertical displacement, were measured. Radiological outcomes were classified using Matta/Tornetta criteria. Subgroup analyses were performed according to fracture type (A/B vs C), presence of sacral or pubic fractures, and bilateral involvement.

**Results:**

Posterior diastasis decreased from 2.54 ± 2.09 cm preoperatively to 0.30 ± 0.67 cm postoperatively. Anterior diastasis improved from 2.54 ± 3.00 cm to 0.35 ± 0.51 cm. Vertical displacement was reduced from 1.18 ± 0.95 cm to 0.36 ± 0.52 cm. Excellent or good radiological outcomes were achieved in 89.3% of cases. No significant differences were observed between fracture types or injury patterns. The complication rate was low, with pin site infection in 7.1% of cases (n = 2) and no observed re-displacement.

**Conclusion:**

The pretensioned pelvic external fixator is a simple and effective technique for achieving immediate radiological reduction in unstable pelvic injuries. It provides reliable correction of displacement, including in type C fractures, and may serve as a valuable tool in both emergency management and as an adjunct to definitive fixation, particularly in resource-limited settings.

**Level of evidence IV:**

Retrospective study.

## Introduction

Research indicates that standard anterior external fixation—whether employing pins in the iliac crest or the supra-acetabular region—can provide stabilization for pelvic injuries but is limited in effectively stabilizing unstable type C pelvic injuries [1–3]. The potential for external fixators to control, reduce, and stabilize injured posterior elements has been a consideration since the advent of pelvic fixation. Over several decades, orthopaedic and trauma surgeons have developed various anterior external fixation configurations aimed at compressing the posterior pelvic elements, with promising results observed in biomechanical studies; however, these systems are often complex and may not be practical for emergency situations [4–7]. Additionally, reducing displacements to facilitate percutaneous fixation in pelvic fractures is considered beneficial from a trauma care perspective, utilizing reduction frames attached to the operating table [8–10].

Most studies focusing on external fixation and related techniques do not report data regarding preoperative displacement and correlates to postoperative reduction achieved for pelvic injuries. Biomechanical and clinical investigations have shown that the pre-tensioned pelvic external fixator (PPEF) offers controlled compression of the posterior elements, contributing to improved load stability and reduction of the pelvis, particularly the posterior structures [11–13].

**Fig. 1:**
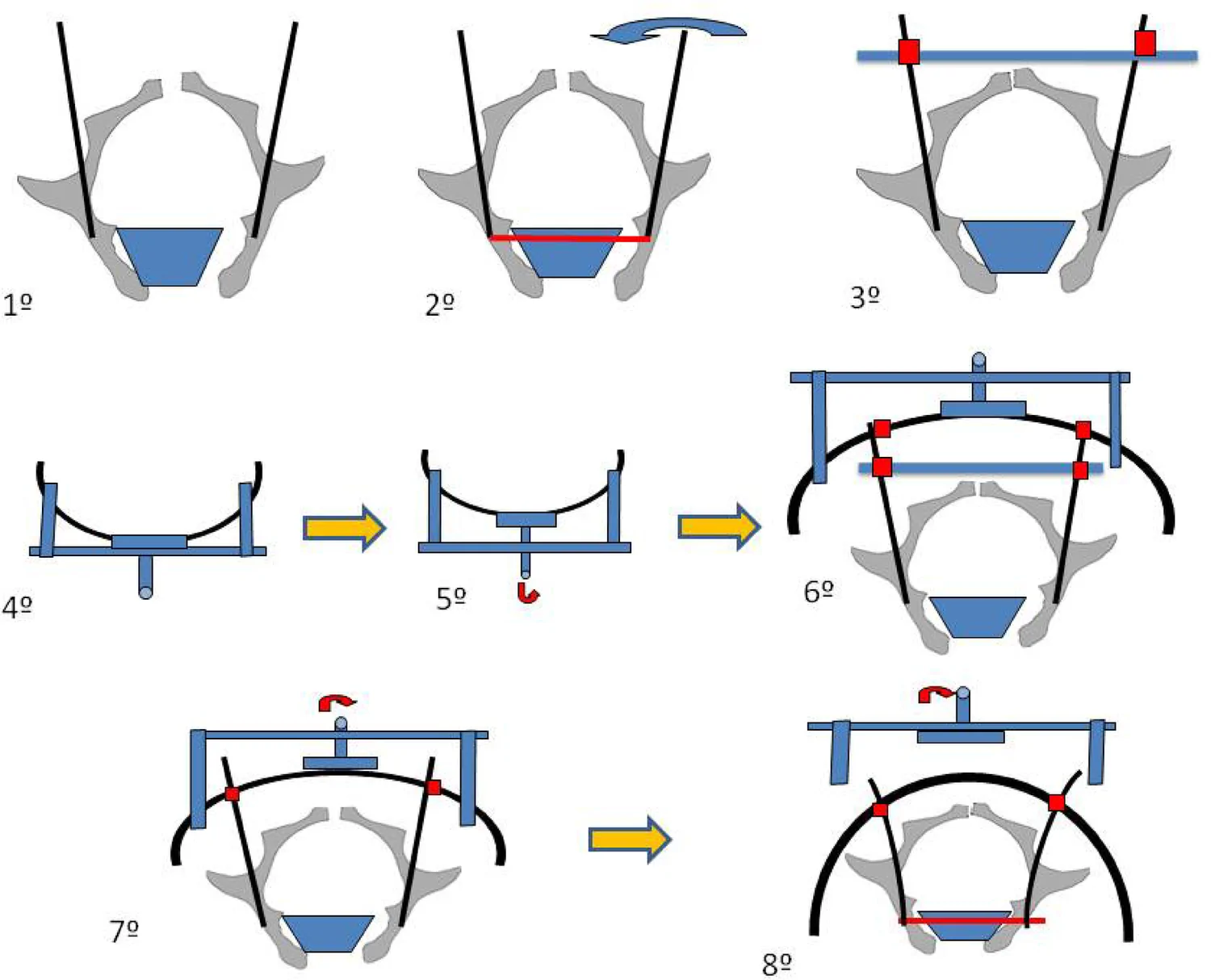
Principle of action of the pre-tensed pelvic external fixator and sequence of reduction: if desired provisional fixation of anterior reduction with a rod (1° to 3°), tense and placement of the pre-tensed curved rod held by the tensor and fixed to the Schanz screws (4° to 6°), removal of the provisional rod (7°). Releasing the tension of the rod will cause that the rod will try to recover its original shape producing anterior and posterior pelvic compression (8°)

The aim of this study was to quantitatively evaluate the radiological reduction achieved using a pretensioned pelvic external fixator (PPEF) in unstable pelvic injuries. This retrospective study assessed the degree of displacement and the reduction by the described surgical technique [12] (Fig. 1) obtained in 28 patients treated with PPEF across a range of clinical scenarios and fracture patterns, including cases with and without vertical instability, associated rami fractures, sacral fractures, and bilateral injuries. In 24 cases, PPEF was used as an emergency stabilization method, whereas in four cases it served as an intraoperative reduction tool.

## Material and method

Between 2012 and 2024, the reduction of the injured pelvis using a pre-tensioned external fixator was analysed in 28 patients with unstable pelvic fractures across varying clinical scenarios. This retrospective study was conducted at a single centre, the Hospital Universitario Virgen de la Victoria. All radiological CT images were anonymized by removing any patient-identifying information (name, hospital ID, and date of birth) prior to analysis and inclusion in this study.

The average patient age was 45.5 years (SD 11.7; median 45; range 16–75), with 78.6% (n = 22) male and 21.4% (n = 6) female. The most frequent mechanism of injury was traffic accidents (53.6%, n = 15), followed by falls from height (32.1%, n = 9) and crushing injuries (14.3%, n = 4). The mean Injury Severity Score (ISS) was 34.8 (SD 10.9; range 10–75). According to the AO/OTA classification, 15 cases were type C, 11 type B, and 2 type A (7.1%). Bilateral involvement was identified in 11 cases (39.3%). Sacral fractures were present in 14 patients (50%) and classified according to Denis as type I in 4 cases (28.6%), type II in 5 (35.7%), and type III in 5 (35.7%), including one sagittal fracture in the sacral body.

### Surgical technique

The PPEF was applied with the patient in the supine position under fluoroscopic guidance. Through bilateral mini-open approaches, two 6 mm Schanz screws using 250- mm × 6-mm apex pins were inserted into the supra-acetabular corridor using the anterior inferior iliac spine as the entry point. For type C pelvic fractures, manual/skeletal traction was employed for vertical displacement reduction. Depending on the fracture pattern, the anterior pelvic ring was reduced by internal or external rotation of the hemipelvis and temporarily stabilized with a straight connecting rod positioned close to the skin. A curved 11-mm carbon-fibre rod (DePuy Synthes, Johnson & Johnson, Raynham, MA, EE. UU.) was then pretensioned using a dedicated tensioning device up to 55mm and then placed and fixed to both Schanz screws with the connexion knots, over the previous one. Once the pretensioned rod had been secured to Schanz screws, the temporary rod was removed. The tensioning device was then gradually released, allowing elastic recoil of the curved rod to generate simultaneous compression of the anterior and posterior pelvic ring. Reduction was confirmed fluoroscopically using AP, inlet and outlet pelvic views before final tightening of the construct [12]. Of the 28 patients, the pretensioned pelvic external fixator (PPEF) was used as a temporary emergency stabilization method in 24 cases (85.8%). In the remaining 4 patients (14.2%), it was used as an adjunct: in 3 cases to facilitate reduction prior to definitive internal fixation, and in 1 case as a definitive stabilization method following failure of internal fixation.

In the emergency group the days until final fixation depended on several variables, but above all on the patient's clinical evolution in the ICU, then definitive internal fixation was performed between 2 and 34 days (mean 12.9 days) after injury. The longest delay (34 days) occurred in a patient with a pin-site infection. The PPEF was maintained as definitive treatment for a Tile B fracture while the infection was successfully treated with intravenous antibiotics. Complications associated with the use of the pre-tensioned external fixator were recorded in 2 cases (7.1%), both corresponding to infections at the pin insertion sites. One of the patients also had a femur fracture treated with an external fixator; all external fixators were removed to perform a definitive two-stage treatment. In the second case, a type B fracture, the fixator was maintained as the definitive treatment. Of the 24 patients treated as emergencies, in 14 patients (58.3%) the sacroiliac screws were inserted before removal of the PPEF, allowing definitive fixation of the posterior pelvic ring while maintaining reduction. Two patients were transferred to another centre, and in the remaining 8 cases (33.3%), the device was removed before osteosynthesis, either for another clinical indication such as spinopelvic fixation or due to a change in strategy to fix the anterior elements first.

**Fig. 2:**
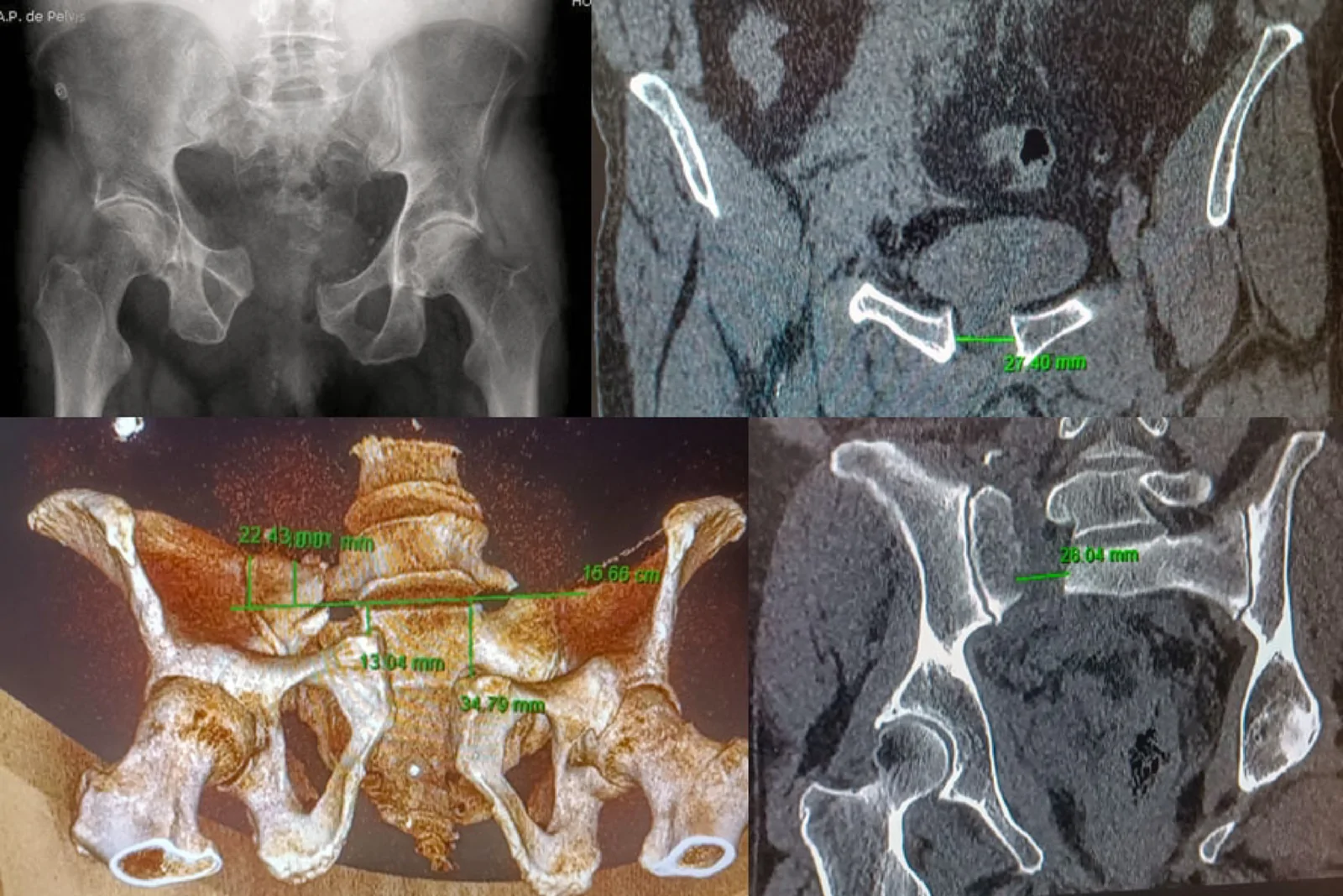
Poly 52 yo male, fall from height with ISS 30. Measurement of pelvic displacements in case 6

**Fig. 3:**
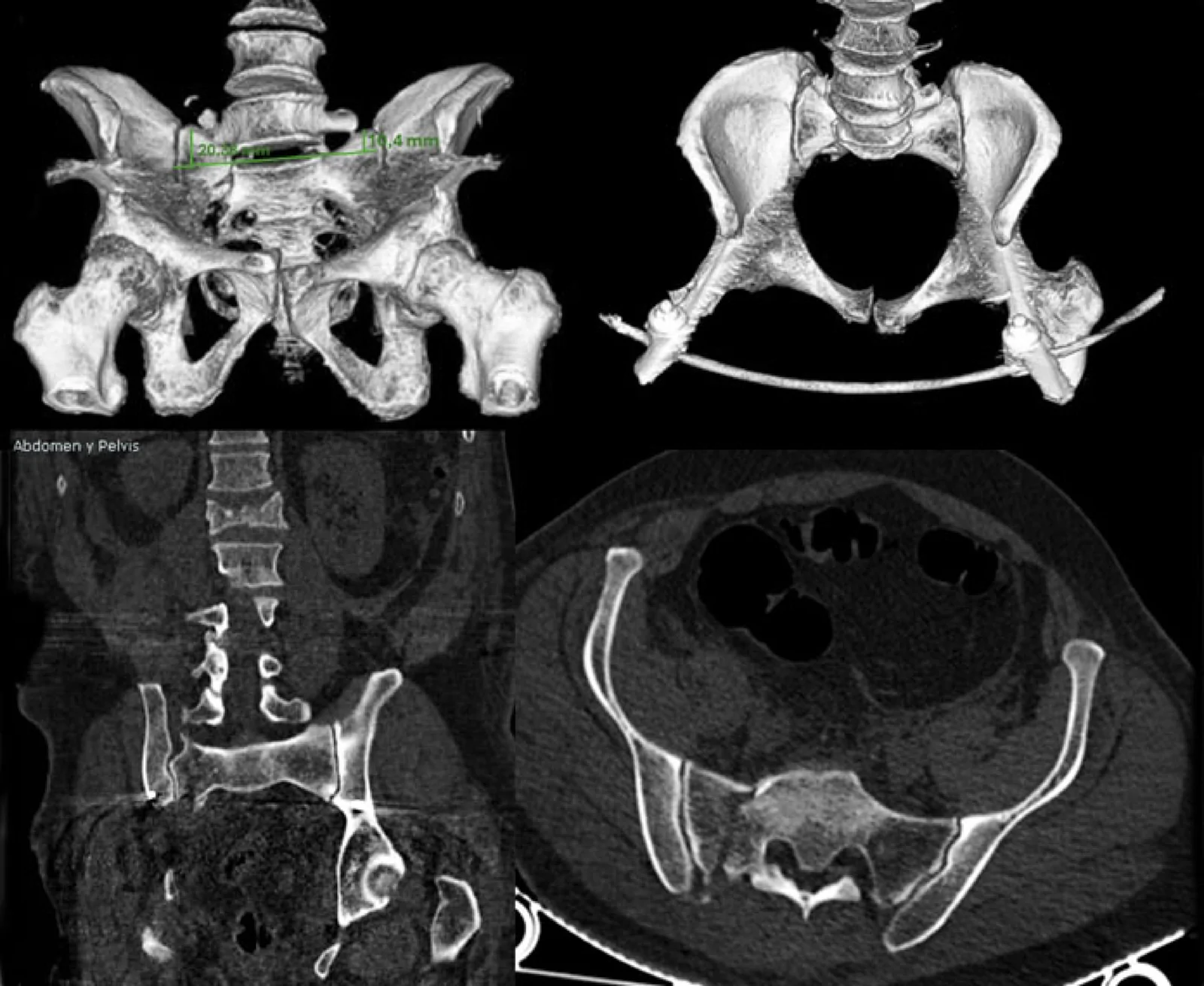
Measurement after reduction with PPEF on case 6; 1 cm displaced vertically and 0 value in symphysis and sacral fracture. This case was classified as a good radiologic result

All measurements were independently performed by two observers, and the mean value of both measurements was used for statistical analysis. Preoperative pelvic displacement or diastasis, as well as reduction achieved after PPEF application, were systematically evaluated using computed tomography (CT) via the Picture Archiving and Communication System (PACS). Anterior and posterior diastasis were assessed on axial and coronal planes parallel to the horizontal plane. To ensure consistency and reproducibility, the maximum displacement measured was selected as the reference value (Fig. 2). Vertical displacement was assessed using three-dimensional reconstructions from a true anteroposterior (AP) sacral view, aligning the S1 vertebral body horizontally. A reference line was established to quantify vertical displacement of the sacral wing or iliac blade. All measurements were recorded in centimetres, with a mean evaluation time of 6.7 days following PPEF placement. At least three CT slices per plane were analysed. A postoperative measurement of zero indicated complete bone-to-bone contact in sacral fractures or full reduction of the sacroiliac joint (Fig. 3).

To evaluate the effectiveness of PPEF under different clinical conditions, patients were stratified according to the Tile classification. Group A/B included stable or partially stable injuries (e.g., open-book lesions or H-type sacral fractures), whereas Group C comprised vertically unstable injuries. Reduction outcomes were further analysed according to the presence of sacral fractures, pubic rami fractures, and bilateral involvement. Radiological reduction quality was classified using modified criteria for external fixation: excellent (pubic diastasis < 1 cm and vertical displacement < 0.2 cm), good (1–2.5 cm and 0.2–0.5 cm), fair (2.5–3.5 cm and 0.5–1 cm), and poor (> 3.5 cm and > 1 cm).

### Statistical analysis

Normality of continuous variables (posterior diastasis, anterior diastasis, vertical displacement) was assessed using the Shapiro–Wilk test. As data were non-normally distributed, results are reported as medians with interquartile ranges (IQR). Between-group comparisons (Type C vs. Types A/B) were performed using the Mann–Whitney U test with Holm correction for multiple comparisons. Within-group pre–post differences were analysed using the Wilcoxon signed-rank test. Effect sizes were calculated using Cliff’s delta for independent samples and rank-biserial correlation for paired data. Categorical variables were compared using χ^2^ or Fisher’s exact test, with odds ratios (OR) and 95% confidence intervals where appropriate. All tests were two-sided with a significance level set at α = 0.05. Statistical analyses were performed using Python (pandas, SciPy).

### Ethics

This study was conducted in accordance with the principles of the Declaration of Helsinki (Fortaleza revision, 2013) and applicable national regulations on biomedical research and data protection. Ethical approval was obtained from the Provincial Research Ethics Committee of Málaga (CEIm Provincial de Málaga), which issued a favourable opinion (reference: SICEIA-2025-003644) on February 26, 2026, for this retrospective observational study. All patients, or their legally authorized representatives, provided written informed consent where applicable. Data were anonymized prior to analysis in accordance with current data protection legislation.

## Results

### Overall reduction results

**Fig. 4:**
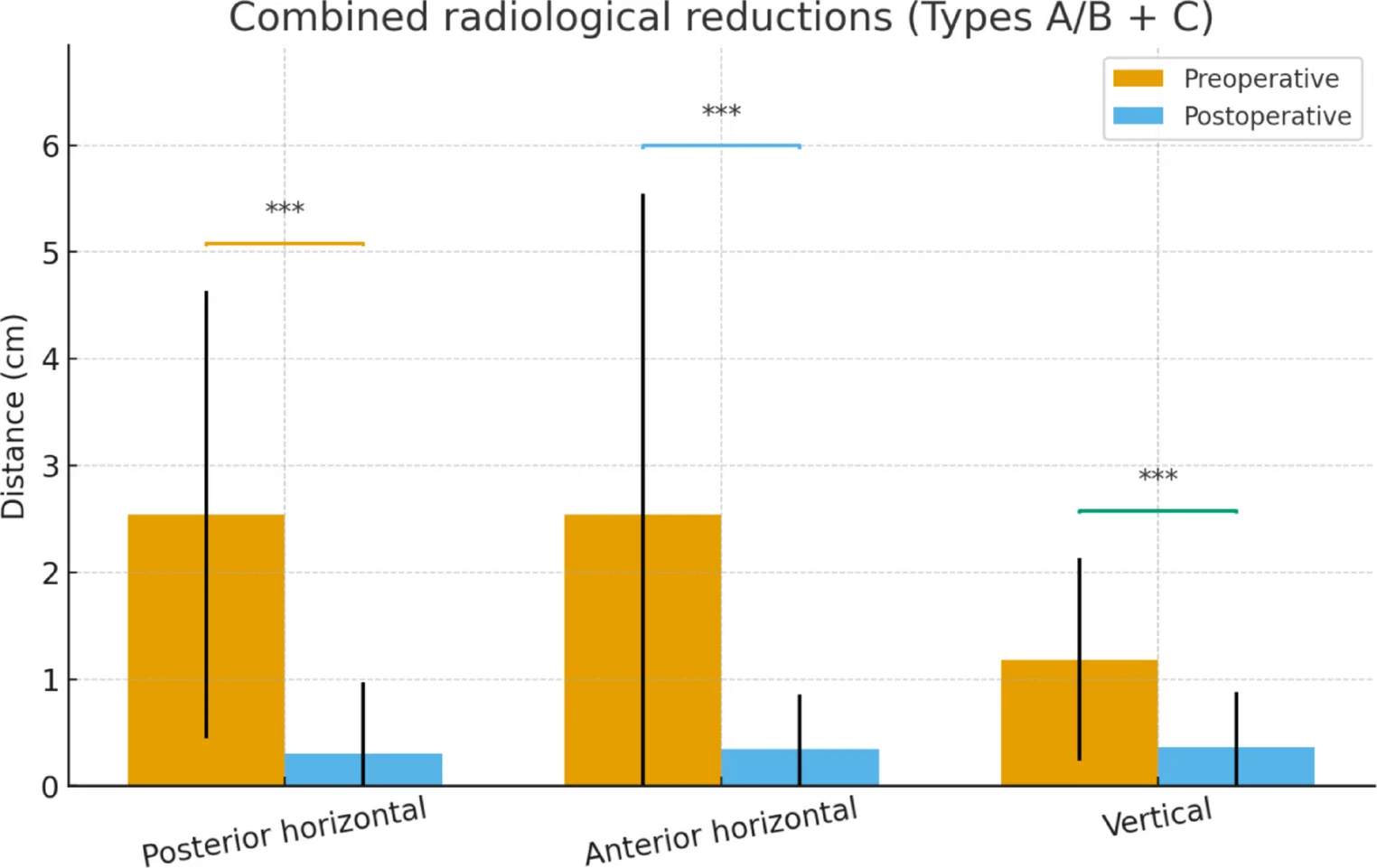
Bar chart of distribution of radiological outcomes according to Majeed’s radiological score in the overall cohort: excellent (n = 16), good (n = 9), fair (n = 2), and poor (n = 1)

Descriptive statistics were compiled for the entire cohort managed with PPEF. Key outcome parameters included posterior diastasis, anterior diastasis, and vertical displacement, each evaluated both prior to and following surgery, in instances involving comminuted sacral fractures accompanied by rami fractures, medialization and internal rotation of the anterior elements were sometimes observed—a phenomenon influenced by the initial reduction technique, particularly during symphyseal reduction done by the surgeon. In the overall cohort (n = 28), a significant reduction was observed across all three displacement axes after application of the Pretensioned Pelvic External Fixator (PPEF). Posterior diastasis decreased from 2.54 ± 2.09 cm to 0.30 ± 0.67 cm (*p* < 0.001), anterior diastasis from 2.54 ± 3.00 cm to 0.35 ± 0.51 cm (*p* < 0.001), and vertical displacement from 1.18 ± 0.95 cm to 0.36 ± 0.52 cm (*p* < 0.001). According to radiographic grading, outcomes were excellent in 16 patients (57.1%), good in 9 (32.1%), fair in 2 (7.1%), and poor in 1 (3.6%) (Fig. 4).

### Subgroup analysis: type A/B fractures

Thirteen patients diagnosed with Type A or B fractures were analysed within this subgroup. Initial stabilization was accomplished using a pelvic binder in 46.2% of cases, while 38.5% did not receive specific interventions. Additional procedures performed included non-therapeutic angiography and diagnostic laparoscopy. Morphological assessment revealed bilateral injury in 46.2% of patients and unilateral injuries in 53.8%. Pubic rami fractures were present in 84.6%, reflecting the predominance of anterior lesions associated with these fracture types. Sacral involvement was observed in 46.2%, indicating posterior ring compromise.

**Fig. 5:**
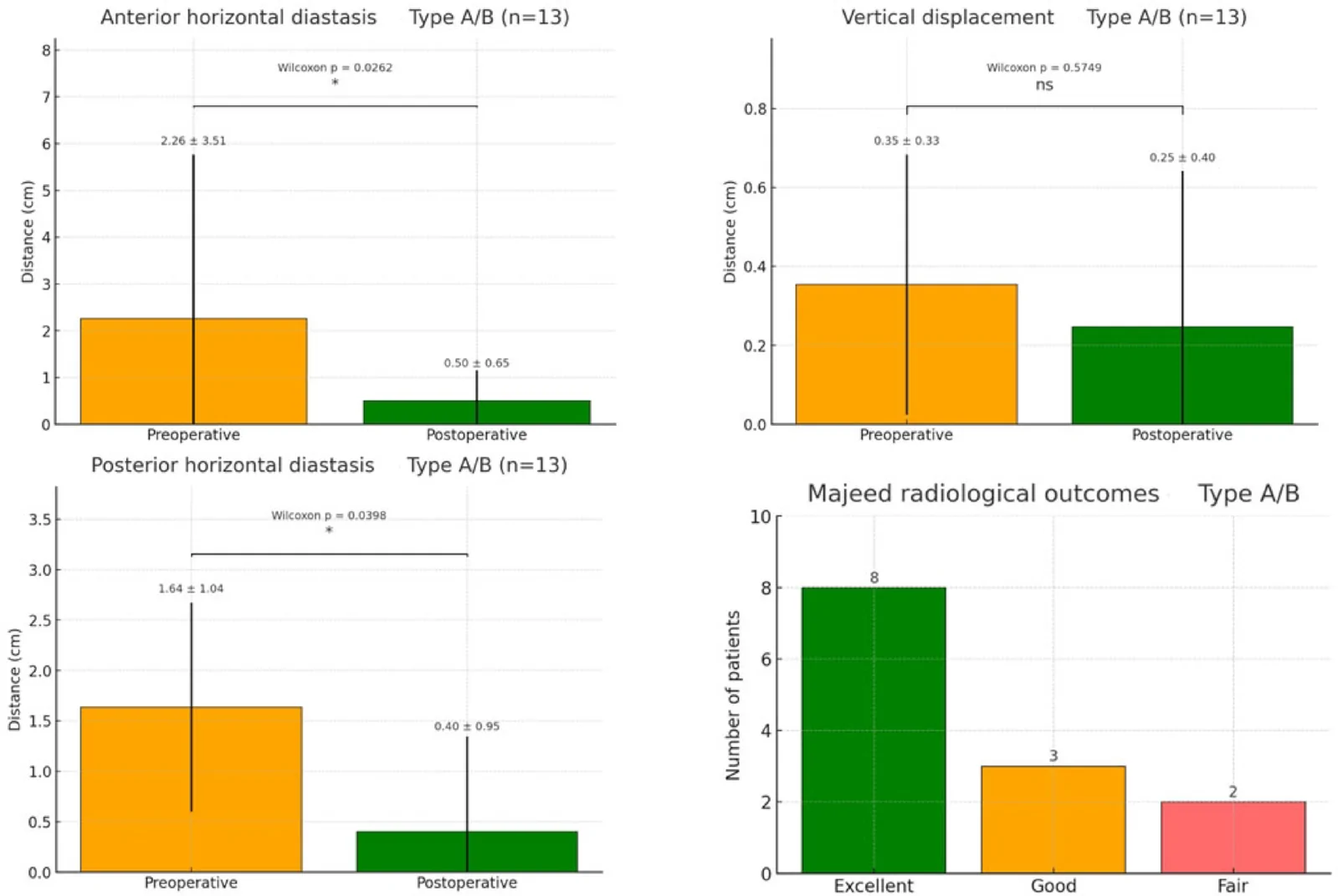
Bar chart group A/B comparing mean preoperative (red, orange, purple) and postoperative (blue, green, cyan) values for posterior diastasis, anterior diastasis, and vertical displacement. A clear reduction in all parameters can be observed after surgery

Among Tile Type A/B fractures (n = 13), posterior diastasis decreased from 1.64 ± 1.04 cm to 0.40 ± 0.95 cm (*p* = 0.0398), and anterior diastasis from 2.26 ± 3.51 cm to 0.50 ± 0.65 cm (*p* = 0.0262). Vertical displacement changed from 0.35 ± 0.33 cm to 0.25 ± 0.40 cm without significant variation (*p* = 0.5749) (Fig. 5).

### Subgroup analysis: type C fractures

**Fig. 6:**
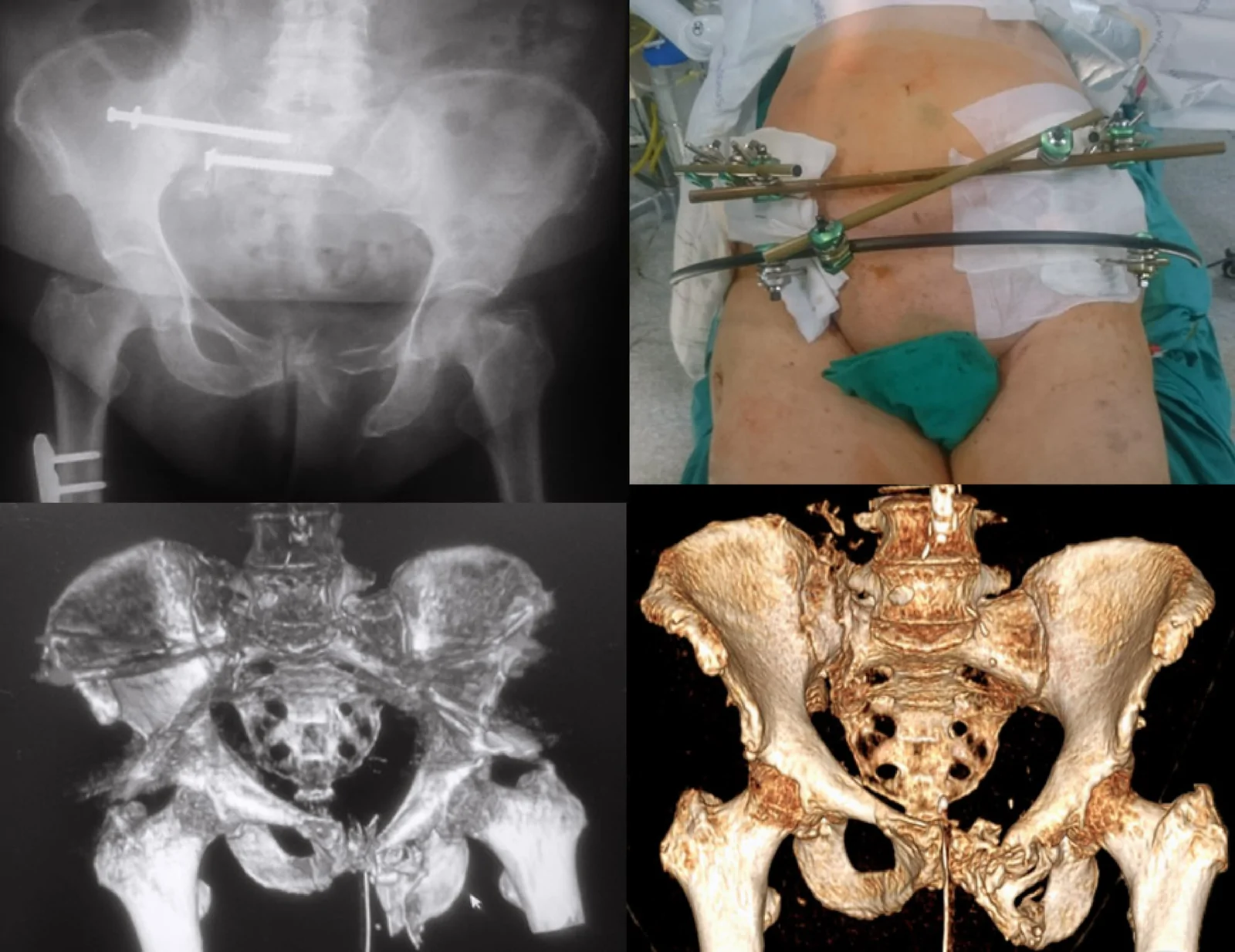
Poly 69 yo female autolysis attempt had a mobilized SI screws after removal of anterior external fixation fixator (**a**). (case 26). Exudative staph. epidermidis + . RMW and rescue, under skeletal traction with PPEF reduce sacral displacement and additional stabilization with iliac external fixation (**b** and **c**). Result after removal of ex fix, with asymptomatic sacral non union

A total of fifteen patients presenting with Type C vertically unstable fractures were evaluated. In four cases, were used as a reduction tools were employed for definitive fixation and in two cases the PPEF was left in place as a complement to other adjuvant fixations (Fig. 6).

Bilateral posterior displacement was identified in 4 patients (25.0%), while pubic rami fractures were noted in 9 patients (56.2%). Sacral fractures occurred in 8 patients (50.0%), representing half of the subgroup. Comparatively, bilateral posterior displacement was less frequent, affecting only 3 patients.

**Fig. 7:**
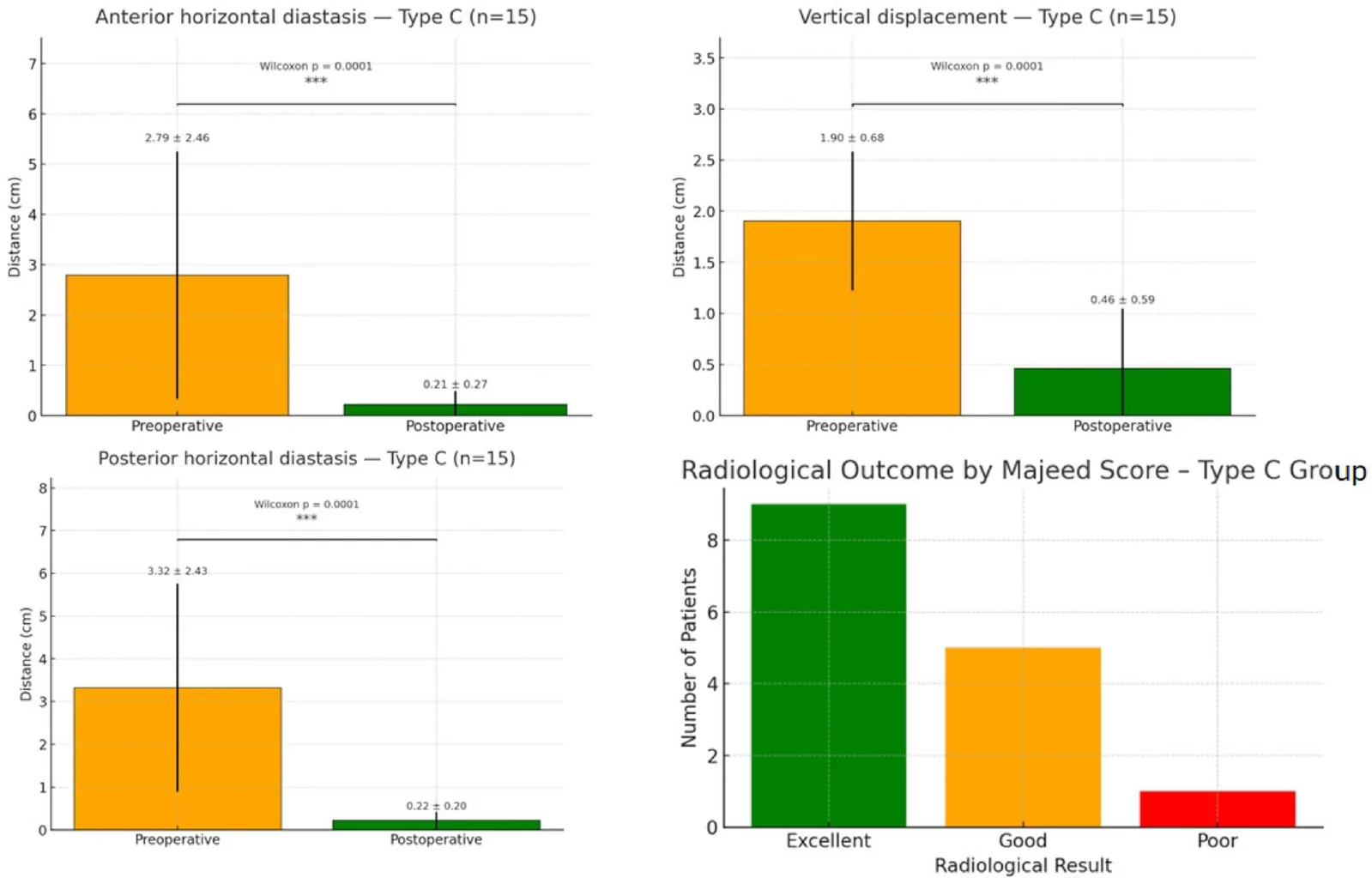
Bar chart group C comparing mean preoperative (red, orange, purple) and postoperative (blue, green, cyan) values for posterior diastasis, anterior diastasis, and vertical displacement. A clear reduction in all parameters can be observed after surgery

Among those requiring emergency surgical intervention, pelvic stabilization was achieved using a pelvic binder in 5 cases (including one ineffective arterial embolization) and a standard external fixator in one case. Five patients underwent surgery without prior pelvic stabilization. In Tile Type C fractures (n = 15), all three dimensions showed significant improvement except for a single outlier case. Posterior diastasis decreased from 3.32 ± 2.43 cm to 0.22 ± 0.20 cm (*p* < 0.001); anterior diastasis from 2.79 ± 2.46 cm to 0.21 ± 0.27 cm (*p* < 0.001); and vertical displacement from 1.90 ± 0.68 cm to 0.46 ± 0.59 cm (*p* < 0.001). Based on radiographic scoring, 93.3% of patients achieved good or excellent postoperative results (Fig. 7). The outlier represented a particularly complex injury pattern, consisting of an acetabular and femoral fracture with irreducible sacroiliac dislocation due to severe sacral comminution following a fall from height.

### Comparison of preoperative displacement and reduction by fracture type

Preoperatively, Type C fractures presented larger displacements than Types A/B, particularly in posterior and vertical planes (*p* = 0.006 and *p* < 0.001, respectively). Mean posterior displacement 3.32 cm vs 1.72 cm, and vertical displacement 1.9 vs 0.46cm.

Postoperatively, no significant between‑group differences were observed across displacement parameters (all *p* ≥ 0.295). Within‑group analyses confirmed significant pre‑to‑post improvements in both cohorts, with larger overall corrections in Type C—especially for vertical displacement (Δ vertical: *p* < 0.001; Δ posterior: *p* = 0.007; Δ anterior: *p* = 0.069). Means anterior displacement type C 0.21 cm vs 0.43 type A/B, posterior displacement type C 0.22 vs 0.14 cm type A/B, and vertical displacement type C 0,46 cm vs 0,09 in type A/B.

Radiographic grading according to Majeed scale indicated that 93.3% of Group C patients had excellent or good results, while one case was classified as poor (6.6%). In Group A/B, 84.6% were rated excellent or good, and two cases were rated fair (15.3%) due to postoperative medialization of the hemipelvis, observed in 2 patients linked to pin placement not crossing the fracture line). The proportion of satisfactory results (excellent + good) did not differ (C 14/15 vs A/B 11/13; *p* = 0.593; OR 3.60, 95% CI 0.32–40.23).

Maximum postoperative residual displacement also showed no difference (C 4.1 [4.0–7.3].mm vs A/B 2.0 [0.0–10.0] mm, *p* = 0.441).

**Fig. 8:**
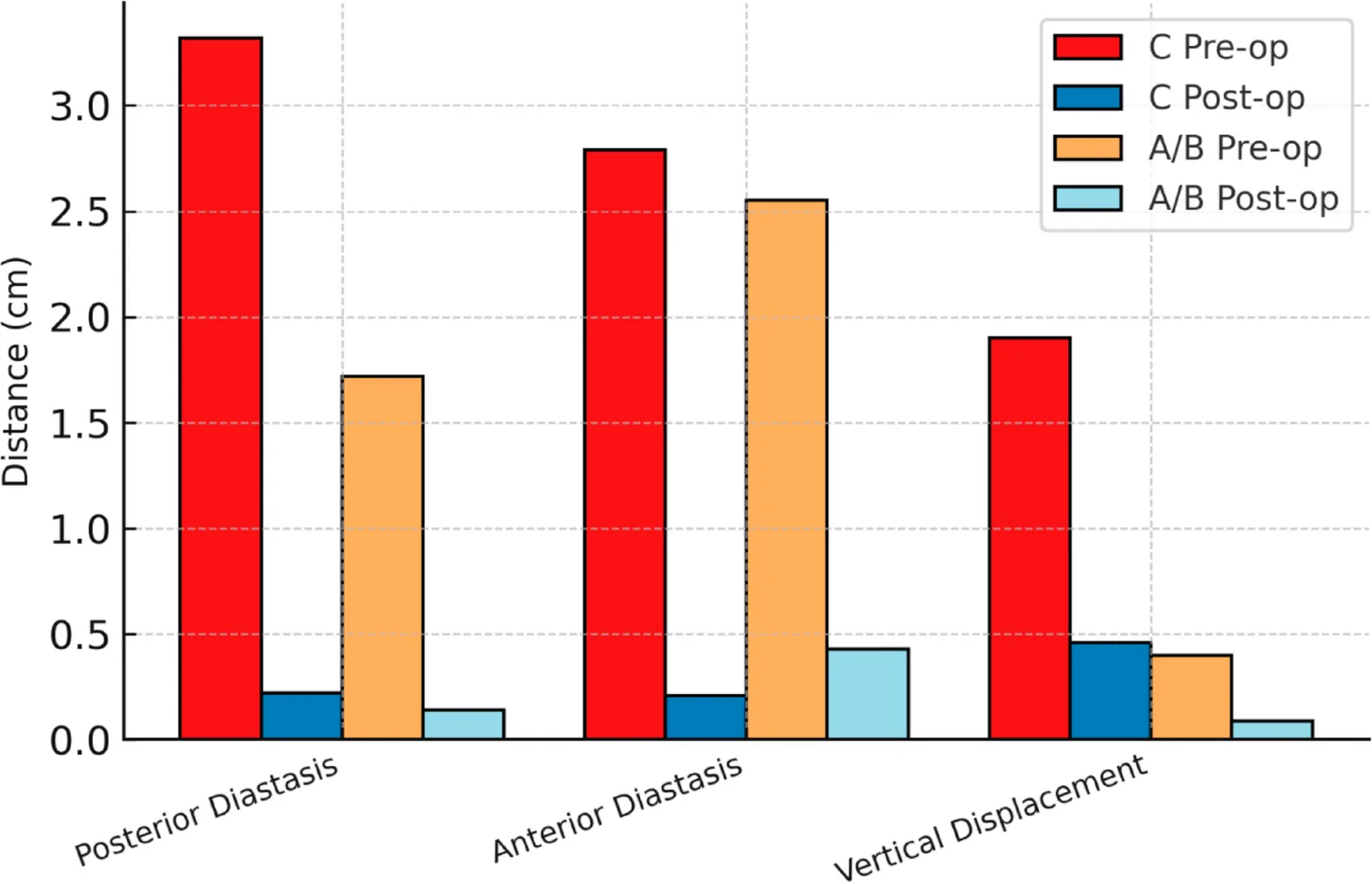
Comparative analysis of mean posterior diastasis, anterior diastasis, and vertical displacement between Group C (red = preoperative, blue = postoperative) and Group A/B (orange = preoperative, green = postoperative). Values expressed in centimetres

The Group C exhibited more severe displacement across all planes prior to treatment. However, postoperative measurements did not show significant differences between groups, suggesting that PPEF achieved similar anatomical reductions regardless of initial fracture severity. Overall, these findings indicate that the PPEF functions as a consistent stabilization method and facilitates three-dimensional reduction in complex pelvic fractures, including multiplanar displaced Type C injuries (Fig. 8).

### Relationship between sacral fracture, bilateral posterior displacement, pubic rami fractures and radiographic result

Although no significant associations were found between specific injury patterns (sacral fracture, pubic rami fractures, bilateral involvement) and the categorical radiographic outcome. The satisfactory result rate (excellent + good) was similar between strata (all comparisons non‑significant).

**Fig. 9:**
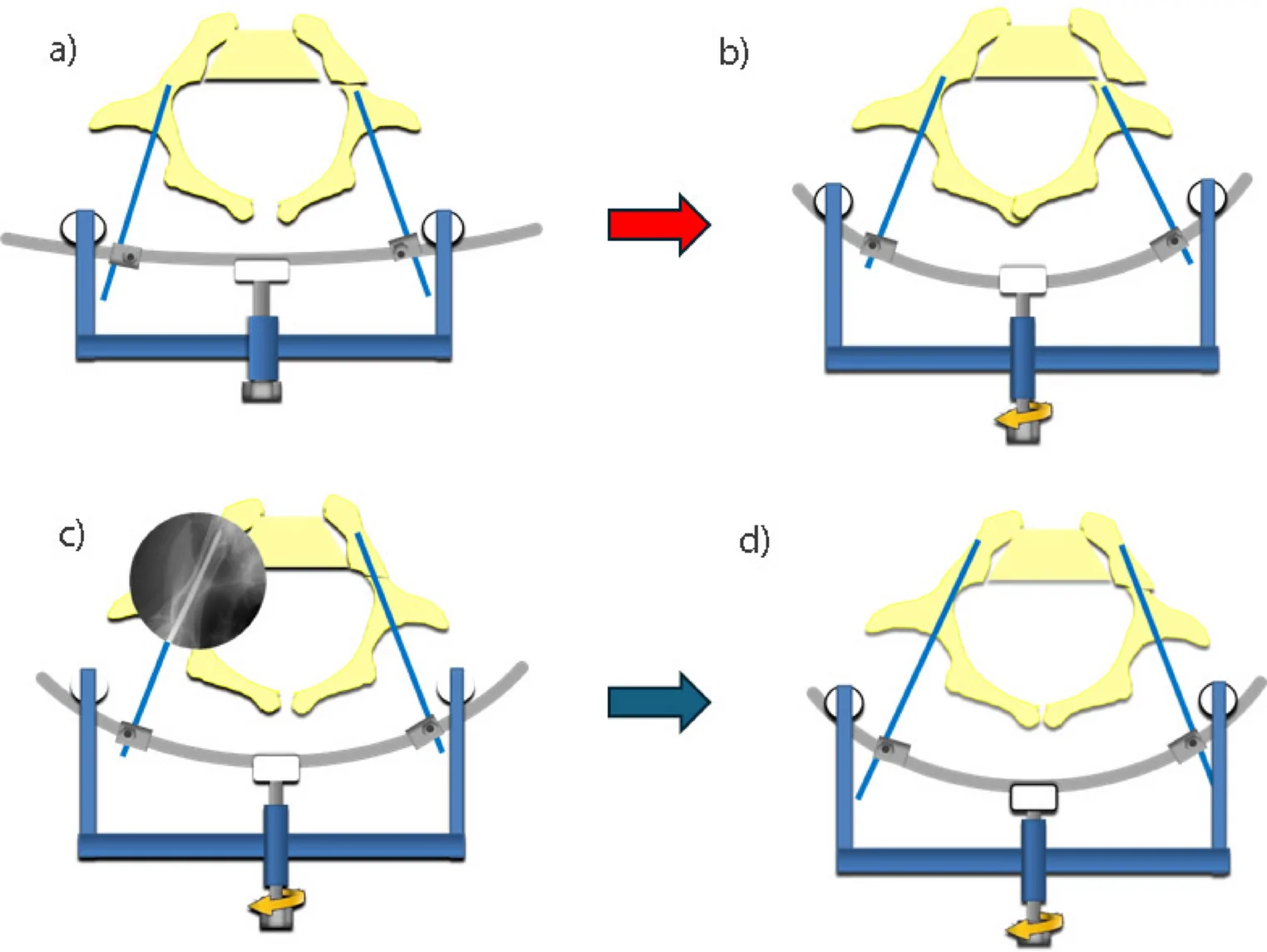
Scheme of undesired displacement in patients with fractures through the ilium. If the Schanz screw does not cross the fracture (**a**), the ilium will medialize when the tension of the bar is released (**b**). If, on the other hand, the Schanz screw crosses the fracture (**cs**), it will not cause this nasty effect, and the ring will be reduced (**d**)

**Fig. 10:**
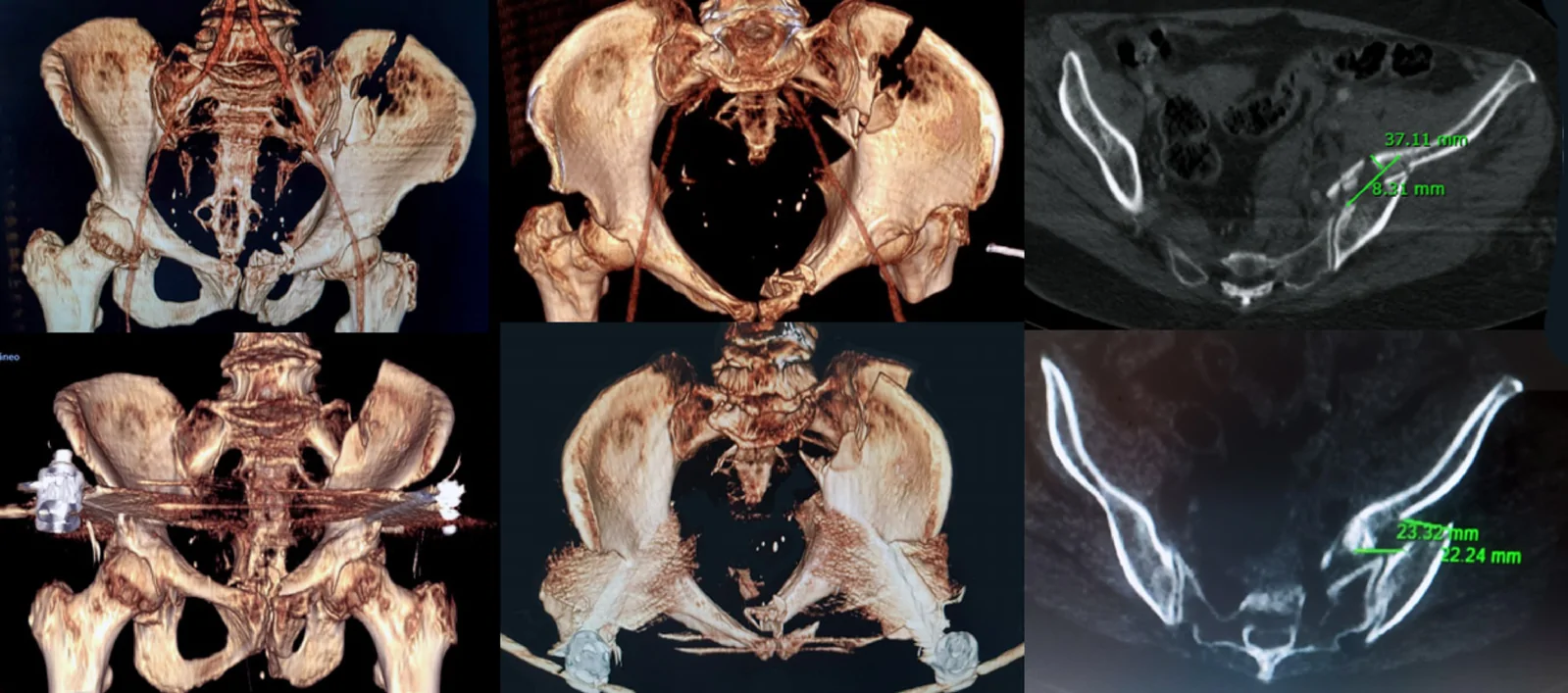
Example of undesirable medial displacement of the hemipelvis in a 73 yo patient with an ilium fracture (case 23) after PPEF radiological result was classified as fair. The patient underwent surgery once hemodynamically stabilized with internal fixation, reducing the displacement without further complications

A potential pitfall of the technique was observed in cases involving iliac fractures in type A/B group. When the Schanz screw does not cross the fracture line, release of bar tension may lead to medialization of the hemipelvis. In contrast, when the screw adequately crosses the fracture, this undesirable displacement is avoided, allowing effective reduction of the pelvic ring (Figs. 9 and 10).

## Discussion

The initial management of high-energy pelvic injuries is widely recognized as a priority, requiring a multidisciplinary approach based on both biomechanical and physiological principles. Pelvic binders represent a rapid, non-invasive stabilization tool essential during patient transport; however, their effectiveness is limited in certain injury patterns, particularly type B fractures and acetabular lesions, and their use beyond 48 h is not recommended [14]. Misapplication may mask severe injuries or exacerbate soft tissue complications. External fixation is commonly advocated upon hospital arrival when hemodynamic instability is present [15]. In our series, 12 patients (50%) who received a pelvic binders presented with hemodynamic instability in the context of polytrauma.

External fixation remains a cornerstone in the early management of pelvic fractures, particularly in hemodynamically unstable patients. Its effectiveness in haemorrhage control is based on three main mechanisms: reduction of pelvic volume, stabilization of fracture fragments, and decreased mobility, which facilitates clot formation. Furthermore, it allows access to the abdominal cavity, can be combined with other fixation strategies, and simplifies patient handling [16].

Despite these advantages, conventional anterior external fixation has limited capacity to control posterior pelvic ring displacement, especially in vertically unstable injuries. This limitation has led to the development of alternative configurations and adjunctive techniques [17]. Some external fixators supposed to resolve the problem e.g. Trapezoidal external fixators, as examined by Lindahl et al. [18] have limitations in exclusively treating unstable pelvic ring fractures, evidenced by elevated rates of malunion (58%), loss of reduction (57%), pin site infection (24%), nerve injuries (2%), and persistent pain (92%). These findings underscore the importance of robust posterior ring control in such injuries.

On the other hand, radiographic assessment prior to the widespread use of CT scanning was not clearly defined in the literature. Lefaivre et al. [8] in a systematic review analysed 31 studies (1,284 cases) reporting radiographic outcomes after surgical treatment of pelvic ring fractures. The authors found that only 3 studies used a standardized, reproducible radiographic measurement method, and 13 applied a grading scale (most commonly Matta & Tornetta or Matta/Tornetta). Overall, 78.6% of cases were reported as having “good” or “excellent” outcomes, but variability in measurement methods weakens the ability to compare results or determine superiority of one fixation method over another.

In our study, CT-based measurements allowed precise quantification of both preoperative displacement and postoperative reduction. Using standardized reference points, including the S1 plateau for vertical displacement, we observed that 89.28% of patients achieved excellent or good radiological results in Majeed score it is particularly applicable for evaluating results after external fixation although this score does not specifically quantify posterior diastasis reduction [14].

Previous studies evaluating external fixation have reported variable results depending on fracture type. Stewart et al. [19] in a systematic review, found that type B fractures generally achieved better reductions than type C injuries, which are inherently more unstable and prone to re-displacement. Complication rates exceeded 50%, largely due to pin site infections 36%, and 12.5% in temporary external fixation. Re-displacement was more common in Type C fractures, consistent with their higher baseline instability, while Type B fractures generally achieved better reductions and functional outcomes. In our series cases pin site infection occurred in only 2 (7.6%) one Type C and one Type B which have left definitive with IV antibiotic. No re-displacement occurs in type C patients after the PPEF application was seen. This low complication rate is potentially attributable to the provisional nature of the fixation and the enhanced stability provided by the FEPP in compressing the posterior elements.

Examples of inadequate reduction and re-displacement in pelvic injuries are often observed in settings with resource constraints or limited technical capabilities, where external fixators may be used as definitive treatment. Studies such as those by Nana et al. [20] and Ba et al. [21] have highlighted these challenges, reporting lower rates of satisfactory reduction, particularly in type C fractures. In this context, the PPEF may offer a cost-effective alternative, as it can be applied rapidly using standard equipment and allows repeated pretensioning without requiring specialized devices.

From a cost–benefit perspective, the PPEF offers potential advantages in such contexts: it can be applied rapidly, although a C-arm is necessary it requires no specialized equipment, the tensor can be easily manufactured and—when locally produced or reused, as the bar can be pretensed several time [11]—significantly reduces treatment costs while providing enhanced mechanical stability. Nevertheless, further studies are warranted to assess the PPEF efficacy as a definitive treatment and clinical correlation on acute and definitive treatment. In our series, owing to specific clinical circumstances, two cases were managed definitively following PPEF application, while in two additional cases the device was employed as an adjunctive tool.

Jäckle et al. [22] determined that optimal anatomical reduction of the sacroiliac complex correlates strongly with positive functional outcomes, though their focus was not solely external fixation. Minimally invasive methods for internal fixation remain a key objective for stabilized patients. Haase et al. described the oblique anterior pelvic external fixator as an intraoperative aid for incomplete sacroiliac joint injuries with external rotation and extension deformities, reporting effective correction of these multiplanar displacements [23].

More advanced reduction systems, such as the Starr Frame or pelvic reduction frames, enable controlled multiplanar correction and have demonstrated excellent radiological outcomes. However, these techniques often require specialized equipment, specific patient positioning, and significant technical expertise, which may limit their use in emergency settings. Similarly, techniques such as the Unlocking Closed Reduction Technique (UCRT) combined with pelvic reduction systems have shown excellent results but are primarily applicable in controlled surgical environments and selected fracture patterns.

Chen et al. [9] employed the Unlocking Closed Reduction Technique (UCRT) for unilateral vertically displaced pelvic ring disruptions (UVDPRD) resistant to conventional methods, utilizing an improved pelvic closed reduction system (PCRS). This yielded anatomical or near-anatomical reductions in 97.6% of 43 patients, with postoperative displacement consistently under 2 mm. According to Matta’s criteria, 86% achieved excellent reduction (< 4 mm). The technique avoided open procedures and facilitated outstanding functional recovery (mean Matta/Tornetta score 94.3). UCRT combined with PCRS provides a reliable, minimally invasive solution for complex UVDPRD cases. This approach requires supine positioning and specialized table adaptations, that are not readily available, making it less feasible in emergency contexts and suitable mainly for unilateral fractures. Ongoing debate surrounds the optimal method for complex posterior pelvic ring fractures—whether isolated ilio-sacral screw fixation or spine-pelvic fixation in the prone position offers superior outcomes [24–26].

In our study, posterior diastasis showed a marked reduction following PPEF application, particularly in type C fractures, In Type C fractures, whether treated acutely or as an adjunct to definitive fixation, a notable improvement was achieved: mean posterior diastasis decreased from 2.05 ± 0.61 cm preoperatively to 0.25 ± 0.21 cm post-FEPP application with results comparable to those reported using dedicated reduction systems. Although some patients underwent prior stabilization, PPEF consistently improved alignment, supporting its role as an effective reduction tool. The long-term maintenance of these corrections warrants further investigation.

This study is limited by its retrospective design, small sample size, and although has a lack of a control group, the cases are controlled by itself a in situ anterior fixation would be fixed with the initial measured displacement. Additionally, no clinical outcomes were assessed, as PPEF was used as a temporary reduction method, focusing exclusively on immediate radiological results. Despite these limitations, PPEF proved to be an effective and versatile technique for achieving controlled reduction in unstable pelvic injuries, with potential value in both emergency settings and resource-limited environments.

## Conclusions

The PPEF (Pretensioned Pelvic External Fixator) is identified as a technique that can be applied to the management of unstable pelvic injuries. The range of indications allows it to be considered for almost universal types of injuries, including cases with sacral fractures, bilateral lesions, or fractures of the pubic rami. Further prospective, randomized multicentre studies are needed to validate these results and refine treatment protocols.

## Data Availability

No datasets were generated or analysed during the current study.
